# Age related changes to the dynamics of contralateral DPOAE suppression in human subjects

**DOI:** 10.1186/1916-0216-43-15

**Published:** 2014-06-16

**Authors:** Ujimoto Konomi, Sohit Kanotra, Adrian L James, Robert V Harrison

**Affiliations:** 1 Auditory Science Laboratory, Department of Otolaryngology-Head and Neck Surgery, The Hospital for Sick Children, 555 University Ave, Toronto, Ontario M5G 1X8, Canada; 2 Department of Otolaryngology-Head and Neck Surgery, University of Toronto, 190 Elizabeth St., Rm 3S-438, R. Fraser Elliott Building, Toronto, Ontario M5G 2 N2, Canada; 3 Department of Otolaryngology, Tokyo Medical University, 6-7-1, Nishishinjuku, Shinjuku-ku, Tokyo 160-0023, Japan

**Keywords:** Outer haircells, Superior olivary complex, Olivo-cochlear efferents, Cochlear inhibition, Aging, Age-related hearing loss, Otoacoustic emissions

## Abstract

**Background:**

The two ears are linked with a neural pathway such that stimulation of one ear has a modulating effect on the contralateral cochlea. This is mediated by cochlear afferent neurons connecting with olivo-cochlear efferents. The monitoring of this pathway is easily achieved by measuring contralateral suppression of otoacoustic emissions, and there is some clinical value in the ability to assess the integrity of this pathway. An important step in an evaluation of clinical utility is to assess any age-related changes. Accordingly, in the present study we measure the dynamics of contralateral DPOAE suppression in a population of normal hearing subjects of different ages.

**Methods:**

Using a real-time DPOAE recording method we assessed contralateral DPOAE suppression in 95 ears from 51 subjects (age range 2–52 years). DPOAE (2f_1_-f_2_; f_2_ = 4.4 kHz; f_2_/f_1_ = 1.22) input–output functions were measured. In response to contralateral broadband noise, dynamic aspects of DPOAE suppression were measured, specifically suppression onset latency and time constants.

**Results:**

An age-related reduction in DPOAE amplitudes was observed. Both the detectability and the degree of contralateral DPOAE suppression were decreased in older age groups. We find an age-related increase in the latency of onset of DPOAE suppression to contralateral stimulation, but no significant change in suppression time-constants.

**Conclusion:**

Olivo-cochlear function as revealed by contralateral suppression of DPOAEs shows some important age-related changes. In addition to reduced emissions (outer haircell suppression) we find an increased latency that may reflect deterioration in auditory brainstem function. Regarding clinical utility, it is possible that the changes observed may reflect an aspect of age-related hearing loss that has not been previously considered.

## Background

Otoacoustic emission (OAE) recording has become an important clinical tool for objective testing of cochlear function, in particular to verify the integrity of the outer haircell system. Distortion product otoacoustic emissions (DPOAEs) [1] are widely used for neonatal screening and other diagnostic purposes in infants, but appear to be less useful in adult testing. In part this is due to an age-related decline in OAEs that has been demonstrated in a number of human and animal model studies [2-8]. The outer haircell system that generates OAE signals can be modulated by activity in olivo-cochlear efferents originating in the superior olivary complex of the brainstem [9,10]. An olivo-cochlear “reflex” can be activated by contralateral acoustic stimulation, which typically inhibits outer haircells, and causes OAE suppression. Many authors have reported on contralateral suppression of DPOAEs in human studies and in animal models [11-14] and have speculated on potential clinical applications [15,16]. It has been suggested that this brainstem reflex may have diagnostic value because the pathways involved include both cochlear afferents and efferents, as well as inner and outer haircell systems. However, contralateral DPOAE suppression appears to have age related deterioration [17-21] that could be a limitation on clinical utility. On the other hand, such deterioration may reveal important age related effects of clinical diagnostic value.

In our previous studies of contralateral DPOAE suppression or modulation [14,16,22,23] we have used real-time recording of DPOAE signals in order to quantify the dynamics of DPOAE changes that result from contralateral acoustic stimulation. In the present study, in normal hearing human subjects, we report on age related changes in these dynamic aspects including the onset latency and time constants of DPOAE suppression. Specifically we report an age-related reduction in DPOAE suppression to contralateral stimulation, and also an age-related increase in the latency of onset of DPOAE suppression.

## Methods

DPOAE levels and the effects of contralateral acoustic stimulation were determined in 95 ears from 51 randomly chosen subjects across a wide age range (2 – 52 years) with no history of sensorineural or conductive hearing loss. We did not formally assess audiometric thresholds and did not exclude subjects because of hearing status because our aim was to assess DPOAE suppression dynamics in the general population. We divided our subjects into five age groups as follows: 1–10 years, 13 subjects (24 ears); 11–20 years, 14 subjects (26 ears); 21–30 years, 8 subjects (15 ears); 31–40 years, 10 subjects (19 ears), > 41 years, 6 subjects (11 ears). This study was approved by the Research Ethics Board at the Hospital for Sick Children, Toronto, and was carried out with the consent of adult participants or parents of children prior to testing.

### Measurement of real-time DPOAE suppression

DPOAEs were recorded in real-time using a custom research device (Vivo 600DPR; Vivosonic, Toronto, Ontario, Canada). The device uses narrow bandpass digital filtering and signal modeling to determine DPOAE levels in real-time with a temporal resolution of <2 ms [23]. All recordings were made in a sound-attenuating room. The DPOAE was determined from the 2f_1_-f_2_ distortion product (f_2_=4.4 kHz; f_2_/f_1_ = 1.22; L_1_ = L_2_ -10 dB). The stimulus levels used for DPOAE recording were adjusted for each subject based on individual input–output (I/O) functions. Thus, I/O functions were recorded with f_2_=4.4 kHz, at levels of L_2_ from 50 - 70 dB SPL, and the stimulus level chosen was that resulting in a DPOAE signal at 0.5 – 0.7 of maximum level [24]. Contralateral stimulation was a 0.5 s duration broadband noise at 50 dB SPL intermittently presented every 1.5 s. This signal was generated by Adobe Audition software (Adobe Systems Inc., San Jose, CA) and presented via an Etymotic Research ER-2 transducer (Etymotic Research, Elk Gorge Village, IL).

### DPOAE data analysis

For each ear we have measured baseline DPOAE levels, and then in response to contralateral stimulation the amplitude of DPOAE suppression, and the dynamic aspects, specifically the onset latency and time constants of suppression. The suppression onset latency was measured as the interval between the onset of contralateral noise stimulus and the initial change point in DPOAE calculated from the intercept of linear regressions plotted through the baseline and first part of the suppression response [14]. Occasionally a very large (by an order of magnitude) DPOAE suppression was observed, caused by a middle ear muscle reflex. These artifacts were excluded from the data before analysis [22]. Tests for statistical significance were made with SPSS software (Sigma Plot 11.2; SPSS Inc., Chicago, IL). Fisher exact test was used in the comparison of presence rate of DPOAE suppression in each age group. Correlation coefficients were determined using Pearson product moment correlation.

## Results

### DPOAE amplitude with age

**Figure 1:**
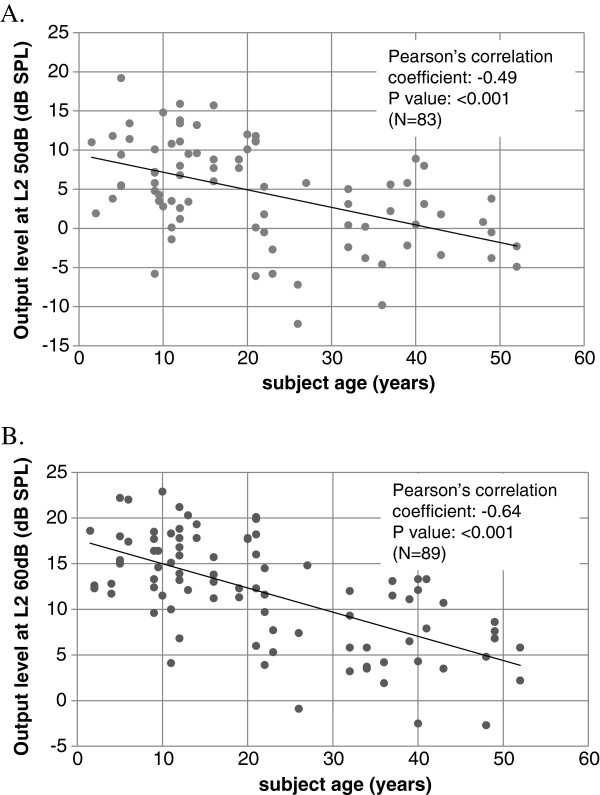
**DPOAE (2f**_**1**_**-f**_**2**_**) amplitude as a function of age.** In the upper plot **(A)** stimulus level L_2_ = 50 dB SPL. In lower graph **(B)** stimulus L_2_ = 60 dB SPL. Pearson’s correlation coefficients and statistical significance values are indicated.

The DPOAE amplitude (f_2_ = 4.4 kHz) as a function of subject age is plotted in Figure 1. In A (upper panel) DPOAE amplitude was determined with L_2_ = 50 dB SPL. In graph B, L_2_ = 60 dB SPL. The data (N = 89 ears) indicates a clear decrement of DPOAE amplitude with age. A linear regression analysis shows a correlation between the DPOAE amplitude and age, and that DPOAE amplitude decreases significantly with age. Thus in plot A (L_2_ = 50 dB) the correlation coefficient = −0.49 (p < 0.001), and in plot B (L_2_ = 60 dB) correlation coefficient = −0.64 (p < 0.001). See Table 1 for details.

**Table 1:** Dynamics (DPOAE suppression onset latency and time constants) and amplitude of contralateral DPOAE suppression in different age groups

**Age group (years)**	**N**	**Age (years)**		**Suppression amplitude (dB)**		**Onset latency (ms)**		**Onset time constants (ms)**	
		**Ave.**	**SD**	**Ave.**	**SD**	**Ave.**	**SD**	**Ave.**	**SD**
***neonates (<3 month)***	***40***	***2.5 (week)***	***-6.7 to 15 (week)***	***3.0***	***-***	***60***	***-***	***-***	***-***
1 - 10	13	6.3	2.9	0.18	0.15	79.2	18.7	379.8	64.8
11 - 20	14	12.8	2.3	0.11	0.08	90.2	28.3	410.1	47.3
21 - 30	8	21.4	0.5	0.17	0.08	109.4	29.4	384.6	113.5
31 - 40	10	34.5	2.9	0.07	0.04	115.8	45.9	415.0	59.1
41 -	6	44.5	4.9	0.05	0.02	137.5	44.5	379.5	115.3
total	15	15.9	11.4	0.13	0.11	94.2	32.0	396.0	65.4

The neonate data is derived from a previous report from our group [16].

### Contralateral DPOAE suppression with age

**Figure 2:**
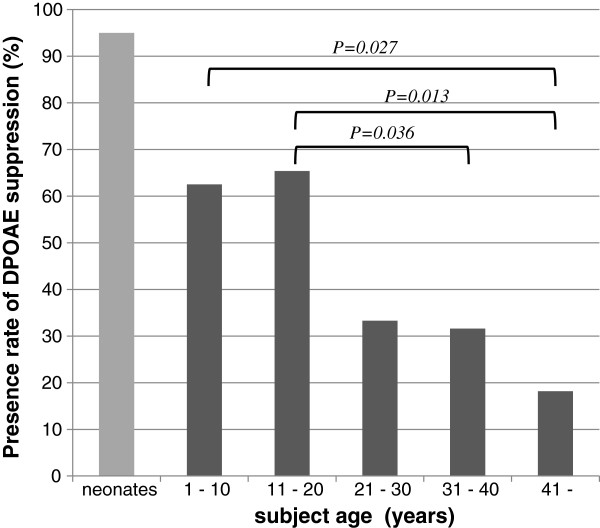
**The presence rate (detectability) of contralateral DPOAE suppression dynamics by age group.** Fisher exact test results are indicated. The neonatal data is derived from a previous report from our group [16].

**Figure 3:**
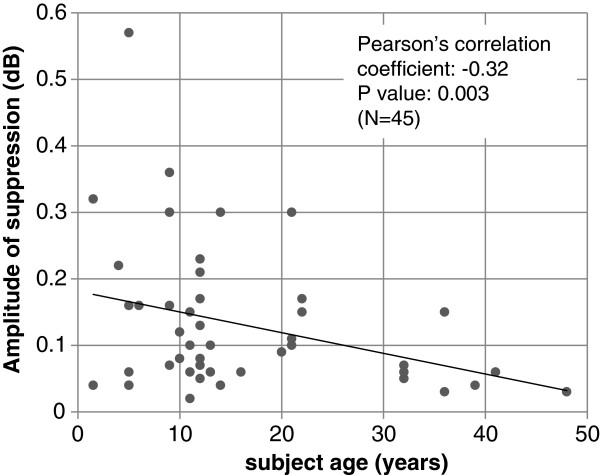
**Amplitude of contralateral DPOAE suppression as a function of age.** Pearson’s correlation coefficients and associated p values are indicated.

Contralateral DPOAE suppression was not reliably observed in all subjects. Overall it could be measured in only 47% of ears. The remaining 53% showed a DPOAE, but there was a lack of OAE suppression to contralateral stimulation. Figure 2 indicates the % presence of contralateral DPOAE suppression as a function of subject age. This is 62.5% in the 1–10 year group; 65.4% in 11–20 year group; 33.3% in the 21–30 year group; 31.6% for the 31–40 year group, and only18.2% in the >41 year group. The differences of those rates in each group were compared by Fisher exact test, and the groups with significant (p > 0.05) differences (1–10 vs. >40; 11–20 vs. 31–40; 11–20 vs. >40) are indicated.Figure 3 shows the amplitude of suppression as a function of age in those subjects where this could be determined. A linear regression analysis shows a low correlation coefficient of −0.32 (p = 0.003) indicating a trend towards less suppression as a function of age. Our data indicate that contralateral DPOAE suppression is more difficult to detect with increasing age either because of reduced DPOAE levels or because of deterioration in olivo-cochlear pathway function with age.

### Age-related changes in DPOAE suppression dynamics

**Figure 4:**
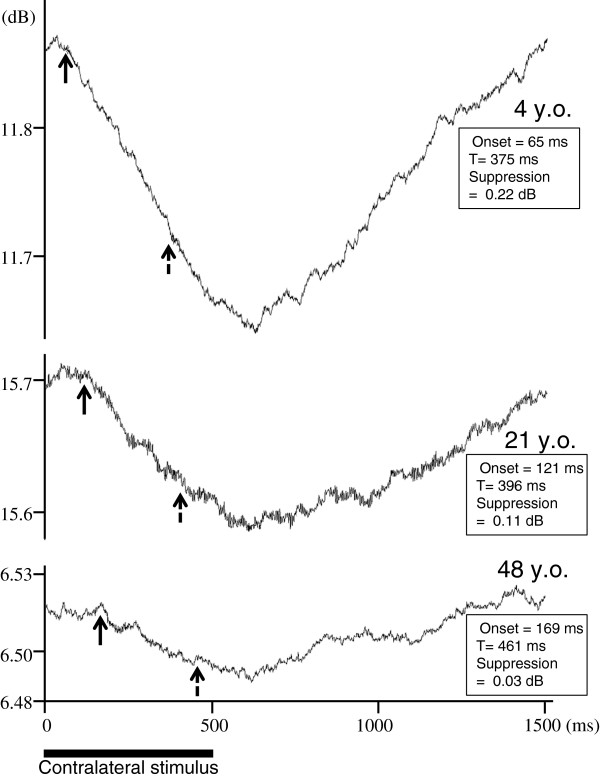
**Real-time derived DPOAE signals from three representative subjects of different ages (4, 21, 48 years).** The lower bar symbol indicates the onset and duration of the contralateral noise stimulus. Arrow symbols mark suppression onset latency, and time constants (data boxes show the exact values).

**Figure 5:**
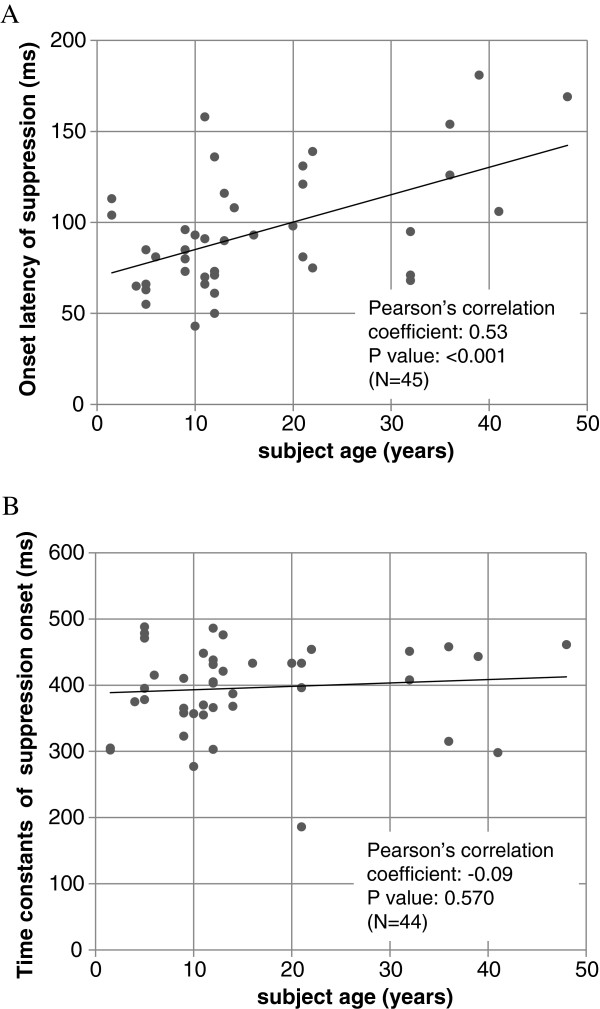
**Dynamic aspects of contralateral DPOAE suppression as a function of age.** The upper plot **(A)** shows onset latency of DPOAE suppression versus age. The lower graph **(B)** shows suppression time constants versus age. Pearson’s correlation coefficients and associated p values are indicated.

Real-time derived DPOAE signals from three representative subjects of different ages (4, 21, 48 years) are shown in Figure 4. The lower bar indicates the duration of the contralateral noise stimulus. The early arrow symbols mark the onset of DPOAE suppression; the second arrow approximates the time constant of DPOAE change (exact value shown in data box). In these examples, as in general, the age-related changes of DPOAE suppression are a decrement of amplitude, and a prolongation of onset latency. The onset latencies of suppression and the time constants of suppression are plotted as a function of subject age in Figure 5. For suppression onset (plot A) there is clearly an increased latency as a function of age indicated by the linear regression analysis (correlation coefficient = 0.53; p < 0.001). For the time constant of suppression (plot B) there is no significant change as a function of age (correlation coefficient = −0.09; p = 0.570). In Table 1, the average (and SD) of suppression magnitudes, onset latencies and suppression time constants are shown for each age group.

## Discussion

### Degradation of DPOAE with age

Previous reports have described a reduction in amplitudes of OAEs with aging [4-8,25]. In addition it has been reported that the rate of spontaneous OAEs is lower in elder subjects [26]. In the present study, DPOAE amplitudes decreased with age similar to that reported in previous work. There has been much discussion about the possible causes of age-related declines and it is likely that many factors contribute. These include loss of sensory cells, degeneration of stria vascularis, and reduction in the endolymphatic potential. Other more central causes of presbycusis such as loss of spiral ganglion cells [27,28] logically do not contribute directly to reduction in outer haircell generated OAEs (although there could conceivable be some indirect effects). Some animal studies of presbycusis have shown that functional hearing loss precedes or is greater than expected from haircell decrements [3,29]. This suggests that perhaps OAE reductions have non-sensory cell origins such as strial degeneration or the degradation of tectorial and basilar membrane integrity.

### Aging of olivo-cochlear function

Recent studies have reported a degradation of contralateral suppression of transient evoked OAE in human with advancing age [30,31]. Oliveira et al. [31] evaluated such changes in subjects from 20 to over 60 years of age and showed that subjects over 40 yrs. had a significant decrement of suppression effect compared with those in their second and third decade. In our study we report a significant reduction in the presence (or detectability) of suppression with age. This is illustrated in Figure 2, in which we have included neonatal data from our previous study [16] where we reported a detection rate of contralateral DPOAE suppression (tested with the same methodology) of 95%. We propose that the age related degradation of contralateral OAE suppression starts from childhood, and is not confined to an elderly population. The corollary to the notion that there is a decline with age is to suppose that olivo-cochlear function is most important during early development. In that respect, a recent study reported that contralateral suppression of OAEs was significantly lower in children with receptive and expressive language delays compared with normal children [32]. It is possible that the medial olivo-cochlear system is important in the development of normal sensory pathways and auditory learning [32,33]. Our results suggest that the olivo-cochlear function, as revealed by contralateral suppression of DPOAEs, is very robust in a neonatal period but declines thereafter.

We note that our mean DPOAE suppression amplitude was relatively small (0.13 dB) compared to some other reports. For example Bassim et al. [34] reported the amplitude of DPOAE suppression by contralateral noise (in 20–30 year olds) to be 1.1 dB. In this case one major difference was the higher intensity (60 dB SPL) and longer duration (>4 s) of contralateral stimulus used compared to 0.5 s in the present study. We use this example to indicate the general difficulty in comparing across studies that use differing OAE measurement methods and stimulation protocols. In our study we find a general reduction in DPOAE suppression amplitude with age that is in agreement with a number of previous reports [17-21,30,31]. The cause of the suppression amplitude reduction can only be speculated on. There are the age-related cochlear changes that we discussed above in relation to OAEs, and in addition the degradation of more central auditory processes should be considered. These include inner haircell synaptic changes, de-myelination of neurons, and functional decline and decrement of cell number in cochlear nucleus [35-37]. Age-related synaptic loss of the MOC efferent innervations [38], the change of calcium regulatory proteins, and neurotransmitter acetylcholine (Ach) [39] have also been reported.

### Age-related change in dynamics of DPOAE suppression

In addition to our own studies [14,22,23] on the dynamics of contralateral DPOAE suppression there have been a number of other reports [34,40-43] however very few describe age-related changes. In mice, Sun and Kim reported that old animals tended to have a smaller adaptation magnitude and longer suppression time constants than younger mice, however they report no statistically significant changes in suppression onset latency [17].

In humans there is still much uncertainty about the actual latency of the olivo-cochlear reflex as revealed by contralateral OAE suppression. Mott et al. reported that contralateral stimulation changes spontaneous OAEs with latencies in the range of 40–200 ms (median = 120 ms); their methodology had a temporal resolution of 40 ms [44]. Lind reported a 40–140 ms latency range for contralateral TEOAE suppression using a system with a 20 ms temporal resolution, respectively [45]. In the present study we use a system with a temporal resolution that can be as low as 2 ms, and have previously reported a DPOAE suppression onset latency of 109 ms in young (20-30 yr) adults [23]. In other reports much lower latencies have been reported. Thus, Maison et al. described a TEOAE suppression latency of less than 60 ms in 25–35 year old subjects [40]. Backus and Guinan reported a very short latency of 25 ms (15-40 ms) for the medial olivo-cochlear reflex in young adults [41]. Other studies have reported on various temporal aspects of the olivo-cochlear reflex [34,42,43], however, comparing these is difficult because of the various OAE recording methods, and protocols used.

It is useful perhaps to dissect out the timing at points along the pathway involved in contralateral suppression of DPOAE as follows: (i) stimulus transduction delay, from acoustic signal onset to the activation of inner hair cells; (ii) afferent neural transmission delay from the cochlea to (anteroventral) cochlear nucleus, to the superior olivary complex; (iii) inter-neuronal connection to cochlear efferent neurons; (iv) efferent neural delay from superior olive to OHCs via the crossed olivo-cochlear bundle; and (v) synaptic and mechanical activation times at OHC level including time for same to be manifest as DPOAE changes. The delay of step (i) is relatively small (<3 ms) and the timing of (ii) is similar to the latency of ABR wave PIII, approximately 6 ms. We will consider delay (iii) later. Regarding to the neural delay of (iv), Fex reported the efferent neural delay is 1.9-3.9 ms in the cat [46]. Since, the length of efferent neurons in human is about 3 times those of cat, the delay of (iv) could be approximated to 10 ms in human. Konishi [47], and Kemp [48] and their colleagues observed (guinea pig) cochlear events (microphonics or OAEs) resulting from electrical stimulation of the crossed olivo-cochlear bundle and report a 10 ms delay which includes OHC post-synaptic events. This suggests that OHC post-synaptic delays are much longer than the cochlear efferent neural delays and that the total delay of step (v) in humans could be 30-50 ms. All of these timing values are, of course, approximations particularly when making extrapolations from animal models. However it is clear that delay (iii), an inter-neuronal processing within the superior olive, adds a significant time factor. There have been reports of age-related functional degradations in brainstem areas including the superior olive [49,50] and we suggest that a significant part of the age related latency increase in DPOAE suppression onset originates at point (iii), the superior olive.

Regarding cochlear events, we report here that DPOAE suppression time constants did not show any significant age related changes (Figures 4 and 5). We interpret this as indicating that OHC mechanical events are not much affected by aging. However we are inclined to interpret our data with some caution. As can be noted in Figure 4, DPOAE suppression did not fully plateau with 0.5 s of contralateral stimulation, thus it is possible that we did not fully measure the overall time constants of DPOAE suppression. Other studies have been more analytical and describe DPOAE suppression or adaptation with a fast and a slow, two-exponent function [34,43]; our data are consistent with time constants reported for the fast component of the suppression, but missing the slow component. Bassim et al. suggest that 10-20 s is required to characterize the slow component [34].

## Conclusions

We report that DPOAE amplitude and DPOAE suppression by contralateral stimulation are degraded with aging, and some dynamic aspects of suppression are also changed with age. Our study was prompted by questions about the clinical utility of DPOAE suppression testing. We conclude that in neonates and young children, contralateral suppression of DPOAEs can be reliably measured, and can potentially inform us about the integrity of OHCs, of cochlear afferent and efferent function and also auditory brainstem mechanisms. We are particularly enthusiastic about our own real-time DPOAE measurement technique because it easily allows the DPOAE suppression dynamics (latency, time constants) to be quantified. We questioned whether such clinical testing was feasible in older subjects and find that the degree and detectability of DPOAE suppression falls off with age. For a clinical test to be widely applicable this may be considered a problem. On the other hand it may be revealing an important parameter of age related hearing loss that we have not been addressing previously.

## Competing interests

The authors declare that they have no competing interests.

## Authors’ contributions

RVH and ALJ formulated the experimental questions, supervised the research and contributed to the manuscript writing. UK and SK conducted all of the experimental testing of subjects, analyzed the data and produced a draft paper. All authors have read and approved of the final manuscript.
